# Molecular Mechanisms of Ischemic Stroke: A Review Integrating Clinical Imaging and Therapeutic Perspectives

**DOI:** 10.3390/biomedicines12040812

**Published:** 2024-04-07

**Authors:** Sana Rehman, Arsalan Nadeem, Umar Akram, Abeer Sarwar, Ammara Quraishi, Hina Siddiqui, Muhammad Abdullah Javed Malik, Mehreen Nabi, Ihtisham Ul Haq, Andrew Cho, Ishan Mazumdar, Minsoo Kim, Kevin Chen, Sadra Sepehri, Richard Wang, Aneri B. Balar, Dhairya A. Lakhani, Vivek S. Yedavalli

**Affiliations:** 1 Russell H. Morgan Department of Radiology and Radiological Sciences, Johns Hopkins School of Medicine, Baltimore, MD 21205, USA; mnabi1@jhmi.edu (M.N.); acho27@jhmi.edu (A.C.); imazumd1@jhmi.edu (I.M.); mkim220@jhmi.edu (M.K.); kchen72@jhmi.edu (K.C.); ssepehr3@jh.edu (S.S.); richardwang@umm.edu (R.W.); aneribbalar@gmail.com (A.B.B.); dlakhan1@jh.edu (D.A.L.); vyedava1@jhmi.edu (V.S.Y.); 2 Department of Medicine, Allama Iqbal Medical College, Lahore 54700, Pakistan; umar.akram2025@gmail.com; 3 Department of Medicine, Fatima Memorial Hospital College of Medicine and Dentistry, Lahore 54000, Pakistan; abeer.sarwars@gmail.com (A.S.); hinasidiki91@gmail.com (H.S.); 4 Department of Medicine, Dow University of Health Sciences, Karachi 74200, Pakistan; ammara.is@gmail.com; 5 Department of Medicine, Wah Medical College, Wah Cantt 47000, Pakistan; javedabdullah507@gmail.com; 6 Department of Medicine, Amna Inayat Medical College, Sheikhupura 54300, Pakistan; ihtishamtarrar@yahoo.com

**Keywords:** ischemic stroke, molecular mechanisms, clinical imaging, radiopharmaceuticals, neuroprotection, ischemic penumbra

## Abstract

Ischemic stroke poses a significant global health challenge, necessitating ongoing exploration of its pathophysiology and treatment strategies. This comprehensive review integrates various aspects of ischemic stroke research, emphasizing crucial mechanisms, therapeutic approaches, and the role of clinical imaging in disease management. It discusses the multifaceted role of Netrin-1, highlighting its potential in promoting neurovascular repair and mitigating post-stroke neurological decline. It also examines the impact of blood–brain barrier permeability on stroke outcomes and explores alternative therapeutic targets such as statins and sphingosine-1-phosphate signaling. Neurocardiology investigations underscore the contribution of cardiac factors to post-stroke mortality, emphasizing the importance of understanding the brain–heart axis for targeted interventions. Additionally, the review advocates for early reperfusion and neuroprotective agents to counter-time-dependent excitotoxicity and inflammation, aiming to preserve tissue viability. Advanced imaging techniques, including DWI, PI, and MR angiography, are discussed for their role in evaluating ischemic penumbra evolution and guiding therapeutic decisions. By integrating molecular insights with imaging modalities, this interdisciplinary approach enhances our understanding of ischemic stroke and offers promising avenues for future research and clinical interventions to improve patient outcomes.

## 1. Introduction

Stroke is traditionally identified as a neurological deficit resulting from an abrupt focal injury of the central nervous system (CNS) caused by a vascular event, which includes cerebral infarction, intracerebral hemorrhage (ICH), and subarachnoid hemorrhage (SAH) [1]. According to the World Health Organization (WHO), each year, 15 million individuals globally experience a stroke. Among them, 5 million succumb to the condition, and an additional 5 million face lasting disabilities, imposing challenges on both families and communities [2]. In over 80% of cases, stroke occurs as a consequence of a transient or permanent decrease in cerebral blood flow secondary to the occlusion of a cerebral artery by either an embolus or a local thrombus [3].

The Stroke Council of the American Heart Association (AHA)/American Stroke Association (ASA) defines ischemic stroke as an episode of neurological dysfunction caused by focal cerebral, spinal, or retinal infarction [1]. Ischemic stroke (IS) immediately triggers a complex molecular and cellular process known as the ischemic cascade. The process sequentially develops several mechanisms as a consequence of the deprivation of blood and oxygen supply to the ischemic core [4]. Patients with ischemic stroke often display the ischemic penumbra, which surrounds the ischemic core and has been identified for up to 48 h. The ischemic penumbra exhibits intermediate perfusion, where cells depolarize intermittently [5,6,7]. Without treatment, the penumbra often progresses to infarction due to the effects of ongoing excitotoxicity, spreading depolarization and post-ischemic inflammation.

Clinical imaging plays a pivotal role in unraveling the molecular mechanisms underlying ischemic stroke. By employing techniques such as computed tomography (CT) and magnetic resonance imaging (MRI), clinicians gain valuable insights into the structural and functional alterations occurring within the brain during an ischemic event. These imaging modalities enable identifying ischemic lesions, assessing blood flow dynamics, and visualizing tissue viability. Integrating clinical imaging with molecular studies offers a comprehensive understanding of the pathophysiological processes at play, aiding in refining diagnostic strategies and developing targeted therapeutic interventions.

## 2. Pathophysiology of Ischemic Stroke

### 2.1. Excitotoxicity

**Figure 1:**
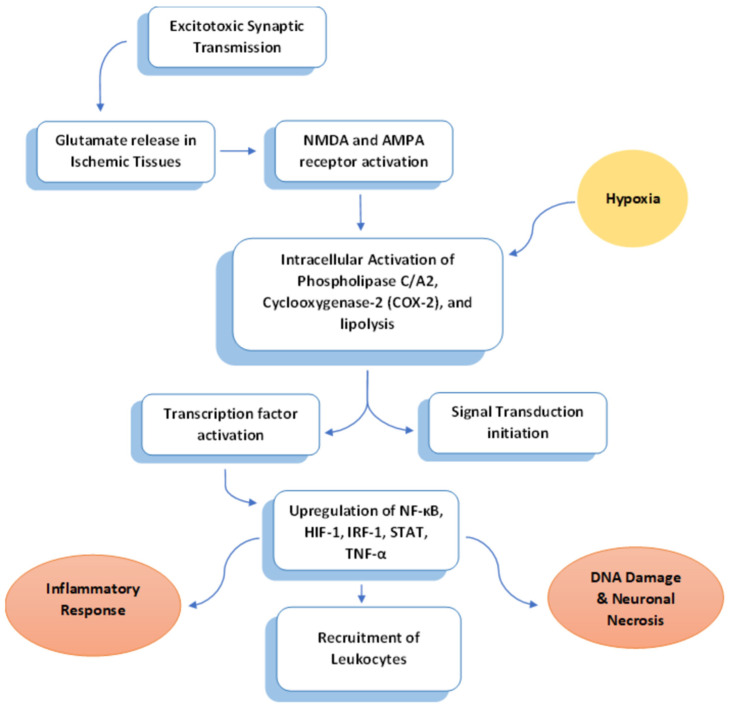
An overview of the mechanism linking excitotoxicity with inflammation during stroke.

Excitotoxic synaptic transmission, facilitated by glutamate through the activation of N-methyl-D-aspartate (NMDA) and alpha-amino-3-hydroxy-5-methyl-4-propionate (AMPA) receptors, is a process where the influx of Na^+^ and Ca^2+^ upon glutamate release depolarizes the membrane (Figure 1). While this mechanism is essential for neuronal plasticity, excessive receptor activation can lead to neuronal death. In ischemic areas, glutamate is released uncontrollably [8]. This unregulated release triggers the glutamate–calcium cascade, resulting in a necrotic lesion, where Ca^2+^-mediated excitotoxicity plays a crucial role in the development of brain infarction [8]. The export of Ca^2+^ from neurons into the extracellular space is intricately tied to energy consumption. In instances of brain energy depletion during hypoxia, there is a passive efflux of K+ from cells, amplifying the entry of Ca^2+^ into neurons. The uncontrolled elevation of intracellular cytoplasmic Ca^2+^ connects glutamate excitotoxicity to biochemical pathways that lead to additional damage, specifically oxidative stress [9].

An increase in Ca^2+^ levels activates phospholipase C/A2, cyclooxygenase-2 (COX-2), and lipolysis, which initiates subsequent signal transduction events involving mitogen-activated protein kinase (MAP kinase), nitric oxide, and lipid peroxidation products (Figure 1). This cascade results in tissue damage and neuronal necrosis [8]. Oxygen free radicals, Ca^2+^, inducible nitric oxide synthase (iNOS), and other hypoxia-induced molecules act as signaling molecules, initiating the inflammatory process within hours of the initial insult [10].

Following tissue damage, transcription factors are swiftly induced in various cell types, such as astroglia, microglia, ECs, leukocytes, and immune cells derived from the periphery (Figure 1). This induction leads to an upregulated expression of inflammatory cytokines and chemokines [11]. Nuclear factor κB (NF-κB), a central player, activates tumor necrosis factor α (TNF-α) and interleukins 1α, 1β, and 6 (IL-1α, IL-1β, IL-6) [12]. Hypoxia-inducible factor 1 (HIF-1) induces vascular EC-derived growth factor (VEGF), intensifying blood–brain barrier leakage and edema [13]. Interferon regulatory factor 1 (IRF-1) stimulates the production of gamma interferon (γ-interferon), activating macrophages [14]. Simultaneously, the activation of signal transducers and activators of transcription (STAT)-1 or 3 results in the overproduction of a platelet-activating factor (PAF), monocyte chemoattractant protein-1 (MCP-1), and intercellular adhesion molecule (ICAM-1) [15].

Prolonged elevation of Ca^2+^ levels triggers the activation of Ca^2+^-dependent effector proteins and enzymes, including endonucleases, phospholipases, lipases, protein kinases, and proteases. These elements have the potential to cause damage to DNA, lipids, and proteins [8]. The exploration and identification of excitotoxic neuronal death modulators are crucial in developing novel strategies to prevent stroke-induced neurological dysfunction.

### 2.2. Inflammation

A decline in cerebral blood flow following a stroke initiates a potent inflammatory reaction within the affected brain tissue. This response is marked by the expression of inflammatory genes, resulting in the local activation and release of diverse cytokines, chemokines, endothelial–leukocyte adhesion molecules, and proteolytic enzymes. These elements propagate inflammatory signals and contribute to the aggravation of tissue damage for several days after the onset of symptoms [3]. Ischemia activates astrocytes, microglia, leukocytes, and ECs, leading to the production of cytokines.

This phenomenon has been observed in experimental stroke models [16] and human patients [17]. Moreover, substantiated evidence indicates cytokine production by activated macrophages [18] and T cells [19] at the site of the evolving cerebral infarction. These cytokines promote the migration of leukocytes from the vascular lumen into the brain tissue and regulate inflammatory and immune responses.

Elevated blood levels of inflammatory markers such as C-reactive protein and fibrinogen are linked to heightened cardiovascular risk in both healthy individuals and stroke patients. Additionally, increased levels of pro-inflammatory cytokines are associated with a more extensive cerebral infarction and a poorer clinical outcome in patients with ischemic stroke. However, the mechanisms through which post-ischemic inflammation contributes to cerebral damage remain not well understood [20].

Both focal and global brain ischemia is associated with the expression of various inflammatory cytokines such as interleukin-1 (IL-1) and tumor necrosis factor-alpha (TNF-α) [21,22,23,24,25], as well as chemokines like IL-8, monocyte chemoattractant protein-1 (MCP-1), RANTES, and IP-10 [26,27,28,29,30]. Concurrently, the upregulation of adhesion molecules (ICAM1 and selectins) induces the recruitment of leukocytes to the vascular endothelium, potentially impacting the survival of damaged neurons [31,32,33,34]. Early expression of adhesion molecules following the insult facilitates the penetration of leukocytes through the blood–brain barrier and plays a pivotal role in this inflammatory process (Figure 1).

In experimental stroke models, ischemic tissue damage has shown reduction with various anti-inflammatory agents. These include antibodies against adhesion molecules [35,36,37], neutrophil depletants [38], inhibitors of pro-inflammatory cytokines [39], and anti-inflammatory cytokines [40,41,42]. The anti-inflammatory cytokines IL-10 and IL-4, predominantly secreted by lymphocytes and monocytes/macrophages, operate in a feedback loop to impede the ongoing production of pro-inflammatory cytokines. Administration of IL-10 in experimental focal brain ischemia models resulted in a decrease in infarct volume, indicating potential neuroprotective effects [41].

### 2.3. Apoptosis

Apoptosis is a highly conserved biological process wherein cells undergo programmed death orchestrated by a specific set of gene products. The condensation of the nucleus and cytoplasm, nuclear fragmentation, and the compact aggregation of nuclear chromatin characterize this process. Additionally, cell shrinkage occurs, ultimately leading to the formation of apoptotic bodies [8]. Apoptosis is a well-documented model for ischemic neuronal death, involving the expression and activation of death-regulating proteins in neurons and non-neuronal cells, and it is caspase-mediated [43].

Cell death following cerebral ischemia can be histologically categorized as either necrotic or apoptotic. Neurons at the ischemic lesion core undergo necrotic death, displaying resistance to caspase inhibitors [44]. In contrast, neurons at the periphery exhibit apoptotic features and can be partially rescued by caspase inhibitors. In the aftermath of a stroke, there is an early upregulation in the gene expression of molecules such as the Bcl-2 family and p53. After the release of proapoptotic molecules such as cytochrome c and apoptosis-inducing factor from the mitochondria, caspases and other genes are activated, which amplifies cell death [45]. The caspase cascade can be initiated through an extrinsic, death receptor-dependent route or an intrinsic mitochondrial pathway [46]. Once activated, effector caspases actively induce the proteolytic degradation of the cell’s structural and functional systems. Ultimately, the activation of caspase-3 is a pivotal event in the execution of apoptosis.

The process of apoptotic cell death critically relies on the activation of caspases by cytochrome c. Caspase-8 or caspase-9 can initiate the well-known pro-apoptotic executioner, caspase-3, contributing to the proteolytic mechanisms within apoptotic cells [47,48]. Several biochemical and immunohistochemical studies have demonstrated the expression and activation of caspase-3 after a stroke. However, there is conflicting information regarding the existence of caspase-3 under ischemic conditions [49]. Furthermore, the expression of p53 can be activated by oxidative stress-induced DNA damage, which can subsequently alter the transcription of various genes, leading to apoptosis induction through caspase-independent apoptosis [50].

## 3. Imaging the Stages of Ischemic Stroke: CT and MRI Correlation

Acute ischemic stroke causes persistent neurologic deficits and brain imaging abnormalities. Imaging and clinical approaches should target extending the therapeutic window for reperfusion treatment with mechanical and pharmacologic thrombolysis to improve treatment strategies. Diffusion-weighted imaging (DWI), perfusion CT, and MRI provide crucial insights into the underlying mechanisms of evolving ischemic stroke, making them valuable tools for enhancing current treatment approaches [51].

### 3.1. Stages of Ischemic Stroke on Imaging

#### 3.1.1. Hyperacute Stage

The term “Hyperacute Stage: Less Than 12 Hours” has gained significant importance with the introduction of IV and intraarterial thrombolytic therapy. Early diagnosis plays a crucial role, as several trials have indicated a therapeutic window for stroke treatment [51]. During the early stages of stroke, cytotoxic edema and thrombus within vessels are visible on imaging [51]. With the advent of newer CT scanners and PACS systems, it is now possible to suspect hyperacute stroke based on CT. Diagnosing and treating acute stroke are facilitated by 4D CT and perfusion CT, which are readily available and quickly acquired. The early signs of stroke on CT result from increased water content in the infarcted area, leading to a lack of visibility of normal anatomical structures [52,53]. These signs include insular ribbon loss, lentiform nucleus obscuration, loss of differentiation between gray and white matter, and sulcal effacement [52,54]. It is important to note that the early signs of stroke may not be visible on CT until 8 h after onset [51]. MRI-DWI has revolutionized stroke imaging by detecting cytotoxic edema within minutes of a stroke. Brownian motion refers to the free movement of molecules in the extracellular space [52,54,55]. An intracellular fluid shift caused by a decrease in ATP, failure of the sodium–potassium–ATPase pump, and anoxic depolarization leads to cell swelling [51]. The changes in the brain during the early stages of an ischemic stroke result in brain cell swelling and the contraction of the space surrounding them, causing a decrease in their movement, which can be detected on a DWI sequence as restricted diffusion (ADC). This condition, known as cytotoxic edema, can be observed within minutes to hours of the stroke and has a sensitivity and specificity of 88–100% and 86–100%, respectively [55]. Fortunately, this stage of infarction is reversible.

#### 3.1.2. Acute Stage: 12–24 h

During the acute stage of injury or damage, cytotoxic edema and intracellular calcium increase further, activating a wide range of enzyme systems, such as proteases, lipases, and nucleases. The production of oxygen-free radicals damages the cell membranes, DNA, and structural neuronal proteins, ultimately leading to cell death [51]. An increased tissue water content causes a lengthening of T1 and T2 relaxation times on MRI scans [51]. T2-weighted imaging shows changes in 90% of patients after 24 h, while only 50% exhibit changes on T1-weighted imaging [51,53]. Additionally, subcortical hypointensity on T2-weighted imaging may result from free radicals sludging of deoxygenated RBCs [51].

#### 3.1.3. Subacute Stage: 2 Days–2 Weeks

When the blood–brain barrier (BBB) is disrupted and swollen cells rupture, there is an increase in extracellular fluid, resulting in vasogenic edema. The development of this process takes approximately 18–24 h and reaches its maximum in 48–72 h [52,53]. During this phase, imaging reveals an increase in edema, mass effect, and possible herniation, depending on the size and location of the infarct [51]. Contrast-enhanced T1-weighted imaging may show gyral and parenchymal enhancement, most pronounced at the end of the first week [51]. It should be noted that signal intensity in the infarcted area remains high on DWI for nearly one week. After that, it decreases, while reduced ADC values peak around 3–5 days, increase thereafter, and return to normal by 1–4 weeks [53,54,55].

#### 3.1.4. Chronic Stage: 2 Weeks–2 Months

During the chronic phase of a brain injury, the restoration of the blood–brain barrier (BBB), subsiding of vasogenic edema, and clearance of necrotic tissue occur. This phase is characterized pathologically and on imaging by local brain atrophy, cavity formation, ex vacuo dilatation of the adjacent ventricle [52,53,55], and visible calcification and deposition of blood products (hemosiderin) on T2 and GRE sequences [51]. Cortical laminar necrosis is a condition that occurs in the later stages of cerebral ischemia, i.e., subacute and chronic stages [51]. Hyperintensity of the cortex is visible on MRI scans between one and three months on T1-weighted and FLAIR sequences, two weeks after infarction [51]. Table 1 outlines the stages of ischemic stroke, detailing the corresponding timeframes and imaging features.

## 4. Advancement in Clinical Imaging Platforms

Researchers have developed a new platform, called the multi-parameter simultaneous fluorescence imaging platform (MPSFL-Platform), to evaluate new drugs for ischemic stroke in real time and in situ. This platform utilizes fluorescence imaging to simultaneously observe three critical indicators of the stroke, namely malondialdehyde (MDA), formaldehyde (FA), and monoamine oxidase A (MAO-A), providing high selectivity and sensitivity. By addressing common shortcomings in drug evaluation methods, this platform allows for more efficient development of drug therapies for ischemic stroke [56].

Fluorescence imaging offers several advantages such as non-invasiveness, real-time monitoring, high specificity, and sensitivity in tracking biological events [56,57,58,59,60,61]. A new MPSFI-Platform was constructed by combining two small molecular fluorescence imaging agents, TFCH and DHMP2 [56]. Multi-color fluorescence imaging materials were meticulously selected to evaluate the biomarkers of ischemic stroke corresponding to these indicators. These materials together formed the MPSFL-Platform [56]. This platform allows for simple operation, real-time, in situ monitoring, and rapid, multi-indicator evaluation of drug efficacy in living cells and mouse brains. This promising new method for assessing efficacy has the potential to revolutionize the field of biomedical imaging [56].

To demonstrate the precision of the MPSFL-Platform in evaluating the efficacy of medication for ischemic stroke, conventional drug efficacy evaluation methods were employed, including evaluation of modified neurological severity score (mNSS), cerebral infarction volume, and brain histopathology. A comparative analysis was conducted between the evaluation results and assessment procedures of the traditional methods and the MPSFL-Platform [56]. The findings highlight that the MPSFL-Platform offers several advantages over conventional drug efficacy evaluation methods for ischemic stroke [56]. Firstly, it enables intuitive and realistic drug efficacy evaluation in living mouse brains, providing real-time and in-situ data in true biological environments without the need to euthanize mice. Secondly, the platform facilitates simultaneous fluorescence visualization of multiple indicators’ levels in real biological contexts, allowing for simultaneous efficacy evaluation with multiple parameters and higher accuracy than traditional methods that can only evaluate one parameter [56]. Lastly, the platform is easy to operate and master, requiring only two materials to be injected into the mice to evaluate drug efficacy. This saves time and resources, and significantly enhances the working efficiency of researchers [56]. Overall, these advantages position the MPSFL-Platform as a highly competitive new method for evaluating the efficacy of ischemic stroke medication. It is worth noting that the MPSFL-Platform surpasses traditional drug efficacy evaluation methods for ischemic stroke in several aspects [56].

## 5. Radiopharmaceuticals in Atherosclerosis Imaging

Atherosclerosis is a chronic and progressive disease that affects the arterial wall due to inflammation, causing acute ischemic events when plaque ruptures and blocks the artery [62]. Vascular inflammation promotes atherosclerosis and its clinical consequences at every stage of the disease, from plaque formation to destabilization [63,64]. Innate and adaptive immune responses trigger active atherosclerotic processes, determining the plaque’s fate [65]. Moreover, systemic inflammation following MI or stroke may accelerate this process. Pre-existing chronic atherosclerosis increases the likelihood of future events [66]. Various other inflammatory processes contribute to plaque vulnerability, such as hypoxia, neo-angiogenesis, and microcalcification [62]. 18F-FDG PET has emerged as an effective imaging tool for detecting inflammation in atherosclerosis, and it has numerous promising clinical applications. This diagnostic tool can provide information about the timing of inflammation in atherosclerosis [62]. Table 2 provides an overview of various imaging techniques for atherosclerosis imaging, detailing their type, invasiveness, imaging contrast agents, and resolution. Each modality offers distinct advantages in resolution, ranging from macroscopic to microscopic scales, catering to diverse diagnostic needs in medical imaging.

Radiopharmaceuticals constitute a category of radioactive substances employed for diagnostic or therapeutic purposes. Despite being typically administered systemically, their biomolecular properties often lead to specific tissue localization [67]. These agents actively emit radiation, posing challenges in storage compared to non-radioactive pharmaceuticals. Diagnostic compounds typically emit beta particles (positrons or electrons) or gamma rays, whereas those emitting Auger electrons or alpha particles (helium nuclei) are generally utilized for therapeutic interventions [67].

In cardiovascular diseases, particularly atherosclerosis, inflammation plays a pivotal role, and numerous biomarkers have been identified due to this process’s chronic and complex nature. This understanding has spurred the development of various radiopharmaceuticals for cardiovascular inflammation imaging, significantly enhancing our insight into biomarker expression during atherosclerosis progression and regression. However, imaging cardiovascular inflammation presents challenges, such as the small size and proximity of atherosclerotic lesions to the blood pool, impacting imaging quality. The intricate pathophysiology of atherosclerotic plaques contributes to low target abundance and dynamic expression, necessitating specificity, sensitivity, radiotracer pharmacokinetics, and appropriate imaging protocols for optimal detection. PET has been preferred among imaging modalities for its high sensitivity, quantitative capabilities, non-invasiveness, and established translational pathways [68,69]. Atherosclerosis development encompasses multiple stages, each featuring unique biomarkers detectable through imaging, providing crucial insights into disease progress and status [70].

For larger arteries like the carotid, iliac, and aorta, 18F-FDG PET imaging is the prevailing method for detecting plaque inflammatory cells, particularly macrophages. In smaller coronary arteries, emerging approaches include 18F-sodium fluoride (18F-NaF) PET and high-resolution intravascular near-infrared fluorescence (NIRF) molecular imaging. These innovative methods enable the imaging of plaque mineralization and inflammation such as protease activity [71].

The application of non-invasive PET imaging, particularly in larger arteries like the carotid, holds promise for future studies. Building on initial insights provided by Marnane et al. [72], investigations into carotid 18F-FDG PET are expected to refine our understanding, identifying high-risk carotid lesions that may necessitate more intensive medical or interventional approaches. This is particularly significant as there is an ongoing debate about the optimal timing for intervention in asymptomatic severe carotid artery lesions, with the potential for biologic data beyond stenosis information to inform such decisions [73].

Further analysis of risk–benefit considerations related to radiation exposure and cost effectiveness is imperative. Beyond its role in assessing plaque inflammation in clinical subjects [74,75,76,77], non-invasive carotid 18F-FDG PET/CT is poised to evaluate novel pharmacotherapies’ anti-inflammatory effects. While new tracers may enhance the sensitivity and specificity of plaque inflammation detection [78], their effectiveness requires validation against outcomes to challenge the established position of 18F-FDG. Table 3 provides a concise overview of molecular imaging agents that illuminate atherosclerosis and their target molecules and clinical applications.

Intravascular near-infrared fluorescence (NIRF) molecular imaging has the potential for high-resolution molecular imaging. It can seamlessly integrate with intravascular ultrasound [79] or optical coherence tomography (OCT) [80] to offer comprehensive molecular–structural imaging of atherosclerosis and stent biology. However, clinical outcome studies are necessary to establish its value despite its promising capabilities. NIRF imaging is still in the early stages of clinical translation and requires further research before it can be widely adopted in clinical practice.

Radionuclide-based molecular agents have been extensively utilized for pre-clinical cardiovascular inflammation imaging. While certain radiotracers have shown promise in clinical investigations for studying human CVD biology, there is a considerable distance to cover in advancing the development of prognostic imaging agents. The goal is to not only sensitively and specifically detect inflammatory biomarkers for clinical translation, but also to foster the progress of precision cardiovascular medicine.

## 6. Netrin-1 and Its Regulatory Effect

Netrin-1, the first purified member in the netrin family, functions as a dual guidance cue. It exhibits either repulsive or attractive properties in axonal pathfinding depending on the specific receptors it interacts with [81]. Evidence suggests that Netrin-1 is crucial in promoting axonal regeneration, synaptic remodeling, neural stem cell migration, and white matter repair across various stroke models. The regulation of angiogenesis, programmed cell death, and neuroinflammation is closely linked to its impact [82,83,84,85,86].

During embryonic development, Netrin-1 exhibits its pro-angiogenic effects, either dependent on or independent of its receptors [87,88]. It stimulates endothelial proliferation, migration, and adhesion to vascular smooth muscle cells [89], thus promoting angiogenesis. This is achieved by increasing endothelial nitric oxide production through DCC-dependent ERK signaling and an endothelial nitric oxide synthase feed-forward mechanism [90]. Netrin-1 also inhibits UNC5B-dependent endothelial apoptosis through DAPK signaling [91,92]. However, in certain instances, it can act as an anti-angiogenic factor, inhibiting endothelial migration and filopodial extension, thereby suppressing angiogenesis when UNC5B is expressed [93,94]. The deletion of the UNC5B gene reverses Netrin-1-mediated angiogenic suppression. Further exploration is warranted to understand the pro- or anti-angiogenic nature of Netrin-1 [95]. Notably, Yang et al. [96] proposed that Netrin-1’s angiogenic role may be concentration-dependent, promoting angiogenesis at low doses while inhibiting it at high doses in vitro. Hence, the observed conflicting outcomes may be attributed to variations in the purity and concentrations of Netrin-1 utilized and the specific types of Netrin-1 receptors expressed on distinct cell types in diverse experimental settings.

The emergence of new capillary blood vessels in the ischemic penumbra enhances blood supply and serves as a structural support for guiding neurons toward the ischemic periphery [97]. AAV-mediated Netrin-1 overexpression further enhances vascular density in the peri-infarct region, potentially reducing infarct size and enhancing functional recovery [98]. Moreover, systemic delivery of the human NT-1 gene by AAV demonstrates the potential to decrease leukocyte accumulation, thereby inhibiting neuroinflammation and minimizing brain parenchymal injury [99]. Collectively, Netrin-1 appears to mitigate neurological decline post ischemic stroke by influencing blood–brain barrier permeability, endothelial function, inflammation, and angiogenesis.

Netrin-1 exerts its neuroprotective effects by activating various anti-apoptotic pathways in response to ischemic stroke. It prevents neuronal loss by suppressing secondary apoptosis, which may occur via its UNC5H2 receptor [100]. Recent studies suggest that Netrin-1 also inhibits endoplasmic reticulum stress and neuronal apoptosis by phosphorylating ERKs through DCC-mediated signaling in both in vivo and in vitro ischemic conditions [101,102]. Moreover, Netrin-1 and DCC co-operatively enhance synaptic formation and axonal regeneration by increasing the activity of phosphorylated JNK1 [103]. Additionally, Yang et al. [104] report that Netrin-1 reduces infarction volume by inhibiting Notch1, a signaling pathway that increases cell susceptibility to apoptosis. Therefore, the anti-apoptotic strategy mediated by Netrin-1 holds significant potential for developing novel stroke therapies.

Microglia, the sentinel cells within the central nervous system’s immune system [105], along with infiltrating macrophages, play a crucial role in orchestrating the immune and inflammatory responses in the brain following ischemic injury [106]. Studies have suggested that these cells exhibit distinct functions and phenotypes during ischemic brain injury, adapting their characteristics to changes in their microenvironment [107,108]. Notably, they manifest two recognized phenotypes: the proinflammatory classical activation phenotype (M1) and the anti-inflammatory alternative activation phenotype (M2). Polarizing microglia towards the M2-like phenotype is crucial to mitigate inflammatory damage after a stroke, as the M2 phenotype promotes tissue repair and regeneration. In contrast, the M1 phenotype releases proinflammatory cytokines that exacerbate tissue injury [109,110].

To explore the role of Netrin-1 in oxygen–glucose deprivation and reperfusion (OGD/R), Yang et al. [111] establish an in vivo model of brain injury induced by middle cerebral artery occlusion and reperfusion (MCAO/R) in rats. The researchers find that overexpression of Netrin-1 significantly decreases the size of the infarction, providing neuroprotection, while the suppression of Netrin-1 increases the extent of the infarction. This neuroprotective effect of Netrin-1 is dependent on AKT activity, as inhibiting AKT reverses the observed benefits. Furthermore, Netrin-1 overexpression improves neurological deficits, reduces apoptosis, and enhances antioxidant activities, outcomes that are compromised in the absence of AKT activity.

In investigating the influence of MCAO (middle cerebral artery occlusion) on NT-1 expression in mice, Lu et al. [112] utilize immunostaining after subjecting mice to 1 h MCAO followed by 1 or 7 days of reperfusion. Their findings reveal increased NT-1-positive cells within the ischemic hemisphere, with a notable concentration in the ischemic boundary zone. This observation suggests that ischemia induces localized NT-1 expression, potentially playing a role in the brain’s reaction to focal ischemia.

## 7. Blood–Brain Barrier Permeability in Stroke

The blood–brain barrier (BBB) restricts the passage of solutes to the brain and is composed of cells and vascular structures held together with tight junctions [113]. A clinical trial visualized BBB changes during stroke using dynamic contrast-enhanced MRI (DCE-MRI), an important tool for various drug discoveries [114]. Studies indicate that ischemic stroke, the most common type of stroke, can cause BBB disruption, which can remain permeable for a few hours to a few weeks after the stroke. Furthermore, BBB in hypertensive patients and patients with other comorbidities may be more prone to damage after stroke [115].

The FDA approved IV-tissue plasminogen activator (IV-tPA) in 1996 as the only drug for the treatment of stroke. However, due to its limitation of use within the first 4.5 h and the potential risk of hemorrhagic transformation, only a small percentage of patients are eligible to receive it [116]. Studies have shown that tissue plasminogen activator (tPA) disrupts tight junctions and basal lamina of the BBB [117], which increases the permeability of the BBB and can result in adverse outcomes. Therefore, it is crucial to explore other drugs and drug targets for the treatment of stroke.

Angiogenesis plays a crucial role in the recovery after a stroke. Statins, known for their cholesterol-lowering effects, have been used as preventative agents after a stroke. HMG-CoA reductase inhibitors, or statins, can increase endothelial nitric oxide synthase and reduce cerebral damage in stroke [118].

Studies conducted on mice have demonstrated that TGF-β can alter the functional barrier of the BBB and affect the expression of tight junction proteins [119]. A study on rats has shown that minocycline can increase the levels of TGF-β [120].

Sphingosine 1 Phosphate (S1P) signaling, a lipid signaling molecule involved in various functions, also plays a role in BBB integrity. Fingolimod, an FDA-approved drug for CNS disorders, targets S1P1, 4–5 receptors [121], and it has shown potential in improving BBB integrity during stroke. An RCT open-label study showed increased efficacy of alteplase when administered with Fingolimod [122]. Various targets, including organic anion-transporting polypeptides (OATPs) belonging to the SLC family of transporters, are being studied for optimal drug delivery to the BBB [123].

Table 4 provides an overview of potential targets for drug discovery aimed at preserving blood–brain barrier integrity during ischemic stroke. Each target represents a specific molecular pathway or cellular component involved in BBB maintenance and can serve as a therapeutic strategy to mitigate BBB dysfunction and improve stroke outcomes.

## 8. Established Pharmaceutical Strategies in Stroke Management

### 8.1. Antiplatelets in Stroke Management

Stroke treatment aims to alleviate current symptoms and prevent recurrence, with systemic thrombolytic therapy being the preferred option for ischemic stroke. However, if the patient has passed the 4.5 h window for thrombolytic therapy, antiplatelet agents should be initiated. Antiplatelet therapy is crucial in preventing stroke by interfering with platelet aggregation [129]. These medications, including aspirin, dipyridamole, ticlopidine, clopidogrel, and cilostazol, work through distinct mechanisms to hinder platelet activation and aggregation, with a significant body of evidence supporting their use either alone or in combination [130,131].

Aspirin, an irreversible inhibitor of cyclooxygenase, has proven effective in doses ranging from 50 to 1300 mg daily for preventing ischemic stroke after a stroke or TIA [132,133,134]. Studies comparing different doses found both high and low doses effective, although higher doses increase the risk of gastrointestinal bleeding [135,136,137,138]. Dipyridamole, which blocks intracellular cAMP signaling related to cyclooxygenase inhibition, works alongside aspirin by blocking different pathways but acts on inter-adenosine diphosphate (ADP) signaling by blocking the surface receptor antagonist (P2Y12) ADP receptor [129]. Research such as the AICLA trial shows that aspirin alone and in combination with dipyridamole have similar efficacy in reducing the risk of ischemic stroke [139,140].

Ticlopidine, as observed in the Canadian American Ticlopidine Study (CATS), reduces the risk of stroke, heart attack, or vascular death by 23% compared to placebo in patients with ischemic stroke [141]. However, it can lead to side effects such as diarrhea, gastrointestinal symptoms, rash, and hemorrhagic complications [142]. Clopidogrel, studied in the CAPRIE trial with over 19,000 patients, was found more effective than aspirin in reducing the risk of ischemic stroke, MI, or vascular death. Yet, its benefit in patients with prior stroke was slightly smaller and not statistically significant [143]. Combining clopidogrel with aspirin lowered the risk of major ischemic events but increased the risk of bleeding [144].

Cilostazol, according to a network meta-analysis (NMA) study, demonstrates the most favorable risk–benefit balance among single and dual antiplatelet therapies for secondary prevention of ischemic stroke or TIA [135]. It reduces the risk of stroke recurrence by 34% compared to aspirin while also lowering the risk of bleeding by 61% [135]. Cilostazol inhibits PDE-3, maintaining platelet aggregation by cAMP activity [145,146]. Its effects may also include delaying the onset of atherosclerosis by inhibiting arterial smooth muscle cell proliferation and maintaining endothelial integrity [147,148,149]. Additionally, cilostazol has anti-inflammatory properties and neuroprotective effects [150,151,152].

The choice of antiplatelet therapy depends on factors such as individual patient characteristics, bleeding risk, and prior stroke history. Each of these antiplatelet agents has demonstrated efficacy in secondary stroke prevention, highlighting the importance of consultation with a healthcare professional to determine the most appropriate treatment regimen for each patient.

### 8.2. Anticoagulants in Stroke Management

Anticoagulants are a class of medications that interfere with the clotting cascade, reducing the formation of thrombi and fibrin networks. While they are crucial in preventing blood clots and potentially dissolving existing ones, their use also comes with the risk of increased bleeding or converting an ischemic stroke to a hemorrhagic one. Commonly used anticoagulants for ischemic stroke include heparin, warfarin, thrombin inhibitors, non-vitamin K antagonists, and vitamin K antagonists [153].

The AHA recommends the use of oral anticoagulants to prevent stroke in patients with cardiovascular risk factors [154]. Several clinical trials have demonstrated the benefits of warfarin for nonvalvular atrial fibrillation. However, for noncardiogenic stroke, antiplatelet agents are preferred over anticoagulants [155]. The WASID trial found that warfarin use, compared to aspirin, increased the risk of hemorrhage and death from vascular or non-vascular causes over a period of 1.8 years [156].

A randomized double-blind study by Kay et al. showed that treatment with low-molecular-weight heparin (LMWH) within 48 h of stroke resulted in better outcomes at 6 months compared to placebo [157]. However, a meta-analysis of 19 randomized controlled trials (RCTs) suggested that LMWH treatment was not superior to standard treatment in reducing all-cause mortality in patients over 70 years old. However, it reduced the risk of deep vein thrombosis in patients under 70. The 2013 CLOTS-3 study demonstrated that intermittent pneumatic compression decreases the likelihood of deep vein thrombosis following both ischemic and hemorrhagic strokes without increasing the risk of bleeding. This treatment was effective regardless of whether background heparin therapy was used [158,159].

Anticoagulants play a crucial role in treating ischemic strokes by preventing blood clot formation and potentially dissolving existing clots. However, their use in hemorrhagic strokes is more limited due to the risk of increased bleeding. Anticoagulants help reduce the risk of recurrent strokes and improve outcomes by preventing clot-related complications.

## 9. Interorgan Interaction in Ischemic Events

Cardiac causes account for the second most significant cause of post-stroke mortality. The subspecialty of neurocardiology investigates the interplay between brain damage and heart injury. The central nervous system manages the sympathetic and parasympathetic functions of the heart. Cardiac arrhythmias pose a risk factor for stroke, and atrial fibrillation may result in neurological vascular disorders. Additionally, myocardial ischemia may increase the incidence of stroke [160,161,162].

Bengel et al. suggested the role of whole-body and molecular imaging in the heart–brain axis in the case of ischemic strokes. The vascular system connects the heart and the brain. The primary mechanisms that cause the interaction between the brain and the heart are (a) RAAS (renin–angiotensin–aldosterone system) due to its involvement in regulating vascular function and its role in homeostasis; (b) the autonomic nervous system that connects both the heart and the brain, since increased sympathetic tone in ischemic stroke can lead to changes in cardiovascular function; (c) immune and inflammatory response that is the key contributor to stroke. Inflammation expresses Tissue Translocator Protein (TSPO), a kDA mitochondrial protein, on microglia and macrophages. A study on male mice showed an increased uptake of 11-C methionine tracer using PET, suggesting astroglial involvement in the heart–brain axis [163]. ACE inhibitors such as enalapril were recommended to decrease the TSPO levels. Overall, imaging modalities play a crucial role in exploring the heart–brain axis crosstalk and should be investigated further to generate targeted responses to improve cardiac and brain function.

Imaging modalities that can evaluate the connection between the brain and the heart during ischemic events include body imaging, functional MRI, and electroencephalography.

## 10. Time-Dependent Excitotoxicity and Inflammation

Hypoxia induces cellular and molecular changes in the brain. The mainstay of treatment for stroke is intravenous thrombolytics with tPA and thrombectomy. Rapid reperfusion following recanalization significantly impacts the molecular outcome. SPECT imaging demonstrates the repair of disrupted perfusion after tPA administration [164].

A retrospective study conducted at a single center on a small patient sample concluded that early reperfusion following recanalization can result in a favorable clinical outcome. However, it does not significantly improve long-term recovery. The proposed mechanism involves targeting IL-6 and glutamate to reduce the inflammatory response observed in stroke. Targeting glutamate receptors can reduce excitatory signals, but it can also lead to decreased signaling in the brain, which can exacerbate damage. The clinical outcome and long-term recovery are affected by serum glutamate and IL-6 levels at admission and 24 h later [165].

A subset of patients demonstrates no clinical improvement despite timely management with rtPA. Andrei et al. have studied this phenomenon and suggest the possibility of stunned brain syndrome [166]. In such cases, neuroprotective agents may be beneficial to patients.

The role of neuroprotective agents as add-ons to improve short- and long-term clinical recovery has been the subject of many clinical trials. An RCT examined the effect of 3-N-Butylphthalide administered 6 h after stroke symptoms treated with rtPA or thrombectomy, which showed favorable long-term clinical outcomes [167]. Clinical trials using Cerebrolysin, a neurotrophic agent with rtPA, demonstrated improved neurological scale outcomes using the National Institute of Health Stroke Scale compared to the placebo group. Another trial showed improved long-term clinical recovery at 12 months using rMS (Rankin modified scale) [168]. The FAST-MAG clinical trial administered magnesium sulfate as a neuroprotective agent with a narrow therapeutic window that did not show treatment benefits.

## 11. Endothelium Dysfunction and Necroptosis after Ischemia/Reperfusion Injury

### 11.1. Role of Endothelium in BBB and Neurovascular Unit

The endothelium, which forms the inner lining of blood vessels, plays a crucial role in the blood–brain barrier (BBB) and the neurovascular unit (NVU). The BBB regulates the exchange of molecules between the bloodstream and the brain parenchyma, safeguarding neural tissue from harmful substances. The NVU, consisting of ECs, astrocytes, pericytes, and neurons, maintains cerebral homeostasis [169].

During ischemic stroke, increased oxidative stress and inflammatory responses lead to EC dysfunction, compromising the BBB’s structural and functional integrity. This dysfunction contributes to increased vascular permeability and edema formation [169].

Following ischemia/reperfusion injury in ischemic stroke, a complex molecular interplay unfolds, where endothelium dysfunction and necroptosis play pivotal roles. The endothelium’s central involvement in shaping the dynamics of the BBB and the NVU becomes evident. The BBB undergoes pronounced disruption during ischemic stroke, resulting in vasogenic edema and hemorrhagic transformation. Early in the ischemic cascade, tight junction protein complexes experience significant alterations, leading to heightened paracellular solute permeability. The NVU plays a pivotal role in influencing the barrier properties of cerebral ECs [126].

Physiologically, the BBB acts as a guardian of CNS homeostasis, protecting brain tissue and regulating ion concentrations, brain water, and electrolyte balance. Tight junction proteins (claudins, occludin, JAMs), adherens junctions mediated by cadherins, and the expression of transporters (ABC and SLC transporters), which are molecular characteristics of BBB ECs, collectively contribute to maintaining barrier integrity. Disruption of BBB integrity is a hallmark of ischemic stroke pathology involving matrix metalloproteinases (MMPs) and integrins. MMPs, particularly MMP-2 and MMP-9, directly compromise the BBB by degrading tight junction proteins, while integrins, which regulate BBB permeability, undergo rapid degradation during ischemic stroke, exacerbating barrier disruption, edema, and inflammation [126].

Understanding the molecular intricacies of the BBB and the coordinated interactions within the NVU provides crucial insights into the endothelium’s central role in ischemic stroke pathology. Targeting specific components of the BBB emerges as a promising therapeutic strategy, offering opportunities to preserve vascular integrity and mitigate the detrimental consequences of ischemia/reperfusion injury. These insights pave the way for the development of novel therapeutic approaches aiming to protect against BBB dysfunction and enhance vascular resilience in the context of ischemic stroke.

### 11.2. Mechanism of Endothelial Necroptosis after Ischemia/Reperfusion Injury

Ischemia/reperfusion (I/R) injury is a significant contributor to necroptosis, a programmed form of necrotic cell death. Reactive oxygen species (ROS) production, triggered by ischemia and subsequent reperfusion, causes oxidative stress and impairs endogenous antioxidant mechanisms. This sets the stage for the induction of necroptosis in ECs [169]. Necroptosis of ECs occurs through the activation of receptor-interacting protein kinase 1 (RIPK1) and downstream effectors such as mixed lineage kinase domain-like pseudokinase (MLKL). The superoxide radical is the primary ROS involved in increased vascular permeability after cerebral ischemia (O^2−^). The process of programmed cell death amplifies blood–brain barrier damage, exacerbating the neurovascular consequences of ischemic stroke [169].

### 11.3. Intervention Using RIPK1-Inhibitor and Infliximab as a Potential Therapeutic Drug

I/R injury poses a significant threat to various tissues, including the endothelium, leading to dysfunction and inflammatory responses. Receptor-Interacting Serine/Threonine-Protein Kinase 1 (RIPK1) is among the key players in orchestrating cell fate decisions following such injury. Mifflin et al. [170] elucidated the crucial involvement of RIPK1 in driving cell death and inflammation during I/R injury. Studies have demonstrated rapid activation of RIPK1 in neuronal and ECs under these conditions, triggering a cascade of events leading to necroptosis and subsequent inflammation. A complex interplay of ubiquitylation, phosphorylation, and other modification events within a multimeric complex known as complex I tightly regulates the activation of RIPK1 [170].

RIPK1 exhibits a dual role, acting as a kinase that promotes cell death and as a scaffold essential for pro-survival NF-κB signaling. The kinase function is associated with inflammation and cell death, while the scaffold function supports postnatal survival. The intricate balance between these functions determines the cellular response to I/R injury [170].

Small-molecule inhibitors of RIPK1 kinase such as Necrostatin-1s (Nec-1s) are widely used in mechanistic studies and animal models. The development of RIPK1 inhibitors, such as GSK′772 for peripheral autoimmune diseases and DNL747 for central nervous system disorders, highlights their potential therapeutic application in mitigating the detrimental effects of RIPK1 activation [170]. Clinical trials investigating RIPK1 inhibitors, including GSK′772, highlight the progress in translating preclinical findings into potential therapeutics. Moreover, Mifflin et al. touch upon the role of TNFα in RIPK1-mediated pathways. This opens avenues for exploring existing drugs targeting TNFα such as infliximab as potential therapeutic interventions to complement RIPK1 inhibition. The combination of RIPK1 inhibitors and existing drugs like infliximab holds promise in addressing endothelial dysfunction and mitigating the consequences of I/R injury [170].

Macrophage migration inhibitory factor (MIF) has been identified as a multifaceted protein that actively promotes receptor-interacting protein kinase 1 (RIPK1)-mediated EC death under conditions of oxygen-glucose deprivation. Surgical trauma induces the expression of MIF in peripheral myeloid cells, and these MIF-loaded myeloid cells adhere to brain ECs after distal middle cerebral artery occlusion (dMCAO), exacerbating BBB disruption [171].

RIPK1, a key mediator of TNFR1 signaling, mediates EC necroptosis and apoptosis in the PIS model. The activation of RIPK1 in cerebrovascular ECs is triggered by cerebral ischemic brain insult, leading to cell death. The study establishes a link between MIF and RIPK1, revealing that myeloid-derived MIF promotes EC apoptosis and necroptosis through an RIPK1 kinase-dependent pathway [171].

Li et al. investigated the use of an RIPK1 inhibitor and a therapeutic drug, infliximab, to explore potential interventions. Genetic deletion of myeloid-derived MIF or pharmacological inhibition of RIPK1 using Nec-1s attenuates BBB disruption and EC death in PIS. Additionally, administration of MIF inhibitor (ISO-1) and RIPK1 inhibitor (Nec-1s) reduces brain EC death and neurological deficits following PIS [171].

The findings suggest that targeting myeloid-derived MIF and inhibiting RIPK1 could be novel therapeutic strategies to mitigate BBB disruption and neurological dysfunction after PIS or other CNS pathologies induced by peripheral inflammation.

## 12. Imaging Ischemic Penumbra and Future Perspectives

### 12.1. Characteristics of Ischemic Penumbra

Ischemic stroke, which constitutes 85% of all stroke cases, results from thrombotic or thromboembolic occlusion in arteries supplying the brain parenchyma, leading to core infarction that is irreversible. The ischemic penumbra, a region of metabolically compromised tissue surrounding the core, is at risk of progressing to irreversible infarction if timely blood flow restoration is not achieved. Energy depletion, neurotransmitter imbalances, and neuroinflammatory responses characterize this penumbra. Cytokine release and microglial activation contribute to secondary neuronal damage, highlighting the dynamic nature of the penumbra and the critical need for timely intervention to salvage tissue [172].

The approach to acute ischemic stroke management has been significantly altered by recognizing the ischemic penumbra as a dynamically evolving and potentially salvageable region within affected brain tissue. The term “penumbra” is unique because it is grounded in the precise functional state of partially ischemic tissue, signifying a nuanced interplay between energy supply and demand. Astrup et al.’s [173] pioneering work highlighted the reversible nature of the ischemic cascade, introducing the concept of functionally impaired yet viable brain tissue. Preservation of this penumbra has become a focal point in reducing morbidity and mortality associated with ischemic stroke [174].

In the molecular tapestry of ischemic stroke, the complexity of the dynamic ischemic penumbra is further emphasized by regional differences in glycogen storage and the protective role of GABAB and adenosine A1 receptors in the white matter. These nuances set the stage for a molecular understanding of vulnerability, emphasizing the need for imaging techniques to capture these complexities [5].

In the context of focal ischemia, researchers have historically defined penumbra as regions characterized by blood flow below the threshold needed to sustain electrical activity but above the level required to maintain cellular ionic gradients, as noted by Obrenovitch et al. [6]. The penumbra represents a delicate balance between energy supply and demand and is a time-limited condition that may evolve towards infarction and potentially spread to adjacent viable tissue. “Misery perfusion”, identified by increased oxygen extraction, acidosis, high glucose utilization with residual ATP, and recurrent spreading depression, contribute to the penumbra’s deterioration, and there is no sustained increase in extracellular glutamate. Electrophysiological changes further exacerbate functional impairment, highlighting the penumbra’s critical importance in clinical contexts, as pointed out by Shimosegawa et al. [175].

Rescuing the penumbra is possible through improving local perfusion and reducing energy demand [6]. The interplay of cerebral blood flow (CBF), oxygen extraction fraction (OEF), and cerebral metabolic rate of oxygen consumption (CMRO2) in the penumbra serves as a key determinant of patient outcomes, [176]. Using sequential positron emission tomography (PET) images in experimental models and stroke patients, Heiss et al. demonstrated that the progressive decrease in CMRO2 and reduction in OEF are predictive indicators of impending infarction. Notably, timely reperfusion before reduction in OEF holds promise in salvaging tissue, highlighting the temporal sensitivity of penumbral assessment [176].

Further insights were gained through studies involving reversible middle cerebral artery (MCA) occlusion in feline models. These experiments emphasized the temporal dependence of penumbral extent on the time of measurement relative to ischemia onset. Early definition of the penumbra in the initial hours of ischemia revealed larger volumes and lower flow values, emphasizing the efficacy of reperfusion strategies during this critical period [176].

### 12.2. Imaging Techniques for Evaluating Penumbra Evolution

Neuroimaging plays a critical role in evaluating the ischemic penumbra by rapidly assessing patients with suspected acute ischemic stroke through a combination of non-contrast head CT, MRI, or both. Although early signs of ischemia may be subtle on CT, especially within the first 3 h, MRI, particularly Diffusion-Weighted Imaging (DWI), offers superior sensitivity in detecting hyperacute ischemia. DWI enables early identification of ischemic tissue viability and aids in determining the extent of the penumbra with a sensitivity ranging from 73% to 100% in the first 3 h [172].

Microstructural alterations are delved into by DWI, offering a molecular perspective on tissue integrity. Perfusion imaging (PI) complements this by providing a real-time snapshot of hemodynamic shifts, aligning with the molecular cascades underlying perfusion changes. MR angiography, through its lens on arterial patency, bridges the molecular and vascular aspects, offering a holistic view of penumbral dynamics [5]. Currently, the PWI/DWI mismatch concept is used to delineate the penumbra, although uncertainties persist due to the dynamic nature of diffusion lesions and methodological limitations [176].

The need for techniques capturing the time-sensitive evolution of the penumbra toward infarction is emphasized. These imaging techniques should provide insights into the evolving state of the penumbra. This implies a focus on methods capable of monitoring changes in blood flow, oxygen extraction, and glucose utilization over time. Real-time imaging modalities become crucial to assess the progression of the penumbra and make informed decisions regarding therapeutic interventions [6].

In the pursuit of evaluating penumbra evolution, Shimosegawa et al. advocate for cutting-edge imaging techniques [175]. Perfusion imaging takes center stage, utilizing perfusion-weighted MRI or CT to offer real-time insights into cerebral blood flow dynamics. DWI emerges as a valuable modality for discerning areas of restricted diffusion, aiding in the precise identification of salvageable tissue within the evolving penumbra. Integrating positron emission tomography (PET) enriches the imaging arsenal, allowing for metabolic assessments that unveil hypoperfused yet viable tissue. Together, these techniques create a dynamic and real-time portrayal of the intricate evolution of the ischemic penumbra [175]. Although PET is considered to be the gold standard, its routine clinical application is limited due to challenges related to accessibility, invasiveness, and exposure to radioactivity [176]. DWI has emerged as a potential alternative to define the ischemic core and perfusion-weighted imaging (PWI) for critically hypoperfused tissue.

Recent advancements can improve the reliability of perfusion assessment in MRI methodologies, such as delay-insensitive deconvolution methods and corrections for tracer delays. However, standardization and quantification challenges persist, and further validation is necessary [176].

Identifying flow thresholds that mark the progression toward infarction is crucial, as emphasized by Astrup et al. [173]. Their investigations centered around the critical ischemic flow threshold, approximately 0.10 mL × g^−1^ × min^−1^, which provides a tangible marker for the transition of the penumbra. Experimental studies involving seizure interruption offer insights into the correlation between electrical and ion pump failure, pivotal indicators in delineating the penumbra’s boundaries. Astrup et al.’s paradigm emphasizes the need for precise imaging tools that can capture the subtle yet decisive changes in the penumbra over time [173].

Traditional imaging modalities such as angiography provide high-resolution definition but cannot discern plaque vulnerability and penumbra evolution. This limitation has spurred the development of sophisticated imaging techniques [174]. PET and single-photon emission computed tomography (SPECT) have emerged as indispensable tools for evaluating carotid plaque vulnerability and brain viability during acute ischemic stroke. These techniques offer high-resolution insights into luminal stenosis and provide crucial information about plaque inflammation, a major predictor for ischemic stroke [174]. Histological studies highlighting plaque inflammation signify the inadequacy of angiography in revealing the complete spectrum of risk factors for ischemic events. Meerwaldt et al.’s data stress the importance of novel imaging approaches in addressing the limitations of traditional methods. By providing a more comprehensive understanding of carotid plaque vulnerability, these advanced imaging techniques are pivotal in refining patient stratification and therapeutic decision making [174].

Several imaging modalities contribute to confirming ischemic stroke diagnosis, excluding mimics, and identifying contraindications to revascularization therapy. CT is widely used for its excellent sensitivity in detecting intracranial hemorrhage (ICH), a strict contraindication to IV-tPA, and endovascular revascularization therapies. MRI enhances sensitivity in detecting ICH with gradient echo sequences and volumetric susceptibility-weighted imaging [172].

### 12.3. Multiparameter Imaging for Accurate Assessment

Recognizing the limitations of singular parameters, researchers have identified the fusion of molecular mechanisms with advanced imaging as the frontier for accurate penumbral assessment. The concept of multiparameter MRI, which integrates DWI, PI, and MR angiography, serves as a molecular canvas capturing the spatiotemporal nuances of the penumbra. This multifaceted approach visualizes and deciphers the molecular underpinnings, providing a comprehensive assessment tool. The emphasis is placed on the proposed paradigm shift towards proof-of-concept imaging studies with infarct growth endpoints, aligning with the evolving landscape of molecular imaging [5].

Advanced neuroimaging techniques, such as CT perfusion and MR perfusion, play a vital role in accurately assessing the ischemic penumbra. These techniques measure blood flow, volume, and mean transit time post-contrast administration, providing insights into the physiological effects of vascular occlusion. Perfusion studies, notably CT and MR perfusion, offer a rapid visual assessment of brain tissue at risk for infarction, aiding in decision making for reperfusion therapy [172]. Moreover, the combination of CT and MRI protocols, leveraging the strengths of each modality, ensures a comprehensive evaluation of the ischemic penumbra. The evolving landscape of neuroimaging research aims to “stop the clock” using advanced technologies, potentially substituting the onset time with neuroimaging evaluation. Metrics such as thrombus length measurement and collateral vessel assessment are emerging as critical factors in refining patient selection for endovascular reperfusion therapy, promising further advancements in enhancing patient outcomes. As technology evolves, time metrics specific to endovascular stroke therapy may be developed, underscoring the ongoing role of neuroimaging in comprehensive patient care [172].

Researchers have integrated PET-derived measurements, encompassing flow values, oxygen consumption, and benzodiazepine receptor ligand flumazenil (FMZ) binding, to provide comprehensive insights into tissue viability. As explored by Heiss et al., the multiparameter imaging approach aims to overcome challenges related to standardization, quantitative measures, and the complexity of data interpretation [176].

## 13. Conclusions

This review highlights the crucial role that clinical imaging plays in unraveling the complex molecular mechanisms that underlie ischemic stroke. Techniques such as CT, MRI, and fluorescence imaging provide invaluable insights into the progression of stroke and the identification of potential therapeutic targets. Moreover, the advancements in radiopharmaceuticals and experimental models offer promising avenues for further exploration. By integrating molecular events with imaging modalities, this interdisciplinary approach enhances our understanding of ischemic stroke and holds significant implications for future research and clinical interventions. Ultimately, this approach aims to improve patient outcomes in this debilitating condition.

## Figures and Tables

**Table 1:** Stages of Ischemic Stroke: Imaging Features and Pathological Processes.

Stage	Timeframe	Imaging Features
Hyperacute Stage	Less Than 12 h	Early signs detectable on CT: Loss of insular ribbon, obscuration of lentiform nucleus, loss of differentiation between gray and white matter, sulcal effacement. MRI-DWI detects cytotoxic edema within minutes, showing restricted diffusion. Cytotoxic edema results from intracellular fluid shift due to ATP decrease, sodium-potassium-ATPase pump failure, and anoxic depolarization. Brownian motion leads to cell swelling, detected on DWI.
Acute Stage	12–24 h	Further increase in cytotoxic edema and intracellular calcium, leading to cell death. Tissue water content increase lengthens T1 and T2 relaxation times on MRI. Changes observable on T2-weighted imaging in 90% of patients after 24 h. Subcortical hypointensity on T2-weighted imaging may result from free radicals sludging of deoxygenated RBCs.
Subacute Stage	2 Days–2 Weeks	Disruption of blood–brain barrier leads to vasogenic edema, increasing extracellular fluid. Imaging shows increased edema, mass effect, and possible herniation. Gyral and parenchymal enhancement visible on contrast-enhanced T1-weighted imaging. High signal intensity on DWI persists for nearly one week; reduced ADC values peak around 3–5 days, then decrease and return to normal by 1–4 weeks.
Chronic Stage	2 Weeks–2 Months	Restoration of blood–brain barrier, subsiding of vasogenic edema, and clearance of necrotic tissue. Imaging features include local brain atrophy, cavity formation, ex vacuo dilatation of adjacent ventricle. Visible calcification and deposition of blood products (hemosiderin) on T2 and GRE sequences. Cortical laminar necrosis visible between one and three months on T1-weighted and FLAIR sequences, two weeks after infarction.

**Table 2:** Comparison of Atherosclerosis Imaging Techniques.

Method	Type of Imaging	Invasiveness	Imaging Contrast Agents	Resolution
MRI	Structural/ functional	Non-invasive	MNPs, Gd- nanoparticles	1–2 mm
PET	Structural/ functional	Non-invasive	18F, 89Zr, nanoparticles	4–5 mm
SPECT	Structural/ functional	Non-invasive	^18^F, ^64^Cu, ^11^C Tracers/^99m^Tc, ^123/124/125/131^I, ^111^In	4–15 mm
CT	Structural/ functional	Non-invasive	AuNPs, iodine-based nanoparticles	1 mm
Angiography	Structural	Invasive	iodine-based	0.16 mm
OCT	Structural	Invasive	ICAM-1-targeting gold nanoshells	0.005–0.02 mm
IVUS	Structural	Invasive	-	0.1 mm
OFDI	Structural	Invasive	Imaging agents with emission wavelengths between 650 nm and 1000 nm	0.01–0.02 mm
NIRF	Functional	Invasive	Indocyanine green (ICG) and methylene blue	0.012 mm

MRI: Magnetic Resonance Imaging, PET: Positron Emission Tomography, SPECT: Single Photon Emission Computed Tomography, CT: Computed Tomography, OCT: Optical Coherence Tomography, IVUS: Intravascular Ultrasound, OFDI: Optical Frequency Domain Imaging, NIRF: Near-Infrared Fluorescence Imaging, MNPs: Magnetic Nanoparticles, AuNPs: Gold Nanoparticles, ICAM-1: Intercellular Adhesion Molecule 1, ICG: Indocyanine Green.

**Table 3:** Molecular imaging agents for visualizing atherosclerosis.

Molecular Imaging Agent	Imaging Modality	Target Molecule/Process	Clinical Applications
Fluorodeoxyglucose (FDG)	PET	Glucose uptake	Assess inflammation in atherosclerotic plaques
18F-Sodium Fluoride (NaF)	PET	Microcalcifications	Assess plaque stability and vulnerability to rupture
99mTc-Annexin V	SPECT/PET	Phosphatidylserine	Detect plaque vulnerability and assess risk of rupture
99mTc-DTPA-mannosyl-dextran	SPECT	Mannose receptor	Target macrophages within atherosclerotic plaques
18F-Fluoromethylcholine (FCH)	PET	Choline uptake	Assess macrophage infiltration in atherosclerotic plaques
68Ga-dotatate	PET	Somatostatin receptor subtype 2	Detect plaque inflammation
Gadolinium-based contrast agents (GBCAs)	MRI	N/A	Visualize plaque morphology and composition

**Table 4:** Potential targets for drug discovery aimed at preserving BBB integrity.

Drug Discovery	Description
Matrix Metalloproteinases (MMPs) [124]	MMPs play a key role in extracellular matrix remodeling and contribute to blood–brain barrier (BBB) breakdown during ischemic stroke.
	Inhibiting MMP activity may prevent degradation of tight junction proteins and basement membrane components, thus preserving BBB integrity.
Vascular Endothelial Growth Factor (VEGF) [125]	VEGF is a potent angiogenic factor that increases vascular permeability and disrupts BBB integrity during ischemic stroke.
	Targeting VEGF signaling pathways or blocking VEGF receptors may attenuate vascular leakage and reduce BBB dysfunction.
Endothelial Cell Tight Junction Proteins [126]	Tight junction proteins, such as occludin, claudins, and zonula occludens (ZO), are critical for maintaining BBB integrity by sealing the intercellular space between endothelial cells.
	Drugs targeting these proteins can strengthen tight junctions and prevent paracellular diffusion of harmful substances into the brain parenchyma.
Inflammatory Mediators [127]	Inflammatory cytokines and chemokines, such as interleukin-1β (IL-1β), tumor necrosis factor-α (TNF-α), and interleukin-6 (IL-6), contribute to BBB disruption and leukocyte infiltration during stroke-induced neuroinflammation.
	Therapeutic agents that inhibit the production or action of these inflammatory mediators may mitigate BBB damage and reduce neuroinflammatory responses in ischemic stroke.
Astrocytic Endfeet [128]	Astrocytic endfeet ensheath cerebral blood vessels and play a crucial role in maintaining BBB integrity through the release of soluble factors and interaction with endothelial cells.
	Targeting astrocyte-derived factors involved in BBB regulation such as aquaporin-4 (AQP4), glial fibrillary acidic protein (GFAP), and astrocyte-secreted neurotrophic factors may enhance BBB stability and protect against ischemic injury.

## Data Availability

No new data were created or analyzed in this study. Data sharing is not applicable to this article.
